# Stroke Propensity Is Increased under Atrial Fibrillation Hemodynamics: A Simulation Study

**DOI:** 10.1371/journal.pone.0073485

**Published:** 2013-09-05

**Authors:** Hyo Won Choi, Jose A. Navia, Ghassan S. Kassab

**Affiliations:** 1 Department of Biomedical Engineering, Indiana University Purdue University, Indianapolis, Indiana, United States of America; 2 Department of Surgery, Indiana University Purdue University, Indianapolis, Indiana, United States of America; 3 Department of Cellular and Integrative Physiology, Indiana University Purdue University, Indianapolis, Indiana, United States of America; 4 Department of Surgery, Austral University, Buenos Aires, Argentina; University of Illinois at Chicago, United States of America

## Abstract

Atrial fibrillation (AF) is the most common sustained dysfunction in heart rhythm clinically and has been identified as an independent risk factor for stroke through formation and embolization of thrombi. AF is associated with reduced cardiac output and short and irregular cardiac cycle length. Although the effect of AF on cardiac hemodynamic parameters has been reported, it remains unclear how the hemodynamic perturbations affect the potential embolization of blood clots to the brain that can cause stroke. To understand stroke propensity in AF, we performed computer simulations to describe trajectories of blood clots subject to the aortic flow conditions that represent normal heart rhythm and AF. Quantitative assessment of stroke propensity by blood clot embolism was carried out for a range of clot properties (e.g., 2–6 mm in diameter and 0–0.8 m/s ejection speed) under normal and AF flow conditions. The simulations demonstrate that the trajectory of clot is significantly affected by clot properties as well as hemodynamic waveforms which lead to significant variations in stroke propensity. The predicted maximum difference in stroke propensity in the left common carotid artery was shown to be about 60% between the normal and AF flow conditions examined. The results suggest that the reduced cardiac output and cycle length induced by AF can significantly increase the incidence of carotid embolism. The present simulations motivate further studies on patient-specific risk assessment of stroke in AF.

## Introduction

Atrial Fibrillation (AF) is a significant independent risk factor for stroke [1]–[8]. The incidence of stroke occurs presumably by the mechanism of blood clot formation in left atrial appendage and subsequent cerebral embolization [3], [9]. The motion of blood clot may not be synchronized with blood flow streamlines depending on flow and clot dynamics. In fact, the discrete phase behavior of the clot in blood flow is determined by a multitude of factors which include initial position and ejection speed of the clot, size of the clot, and dynamics of blood flow.

Although AF has been shown to lead to a broad spectrum of abnormal cardiac hemodynamic parameters [10]–[13], the effect of these hemodynamic perturbations on embolic propensity has not been systematically addressed. Specifically, it is unclear which elements of altered hemodynamic characteristics in AF may attribute to increased incidence of cerebral embolization.

Here, we performed computational fluid dynamics (CFD) simulations to understand the flow trajectories of blood clots in normal and AF waveforms. For each flow condition, trajectories of blood clots with diverse physical properties were tracked with each cardiac cycle. The number of clots that entered the left common carotid artery was tracked and the frequency of clot embolization was compared for the normal and AF flow conditions. The present study provides a computational platform that allows a quantitative analysis of the relation between hemodynamic parameters and blood clot trajectories. Hence, the model can eventually be used for a patient-specific risk assessment of embolic stroke. Furthermore, the current computational approach may be utilized for the design of vascular devices to prevent stroke by deflecting local clot trajectory away from carotid arteries.

## Computational Methods

### Geometry and Computational Domain

**Figure 1:**
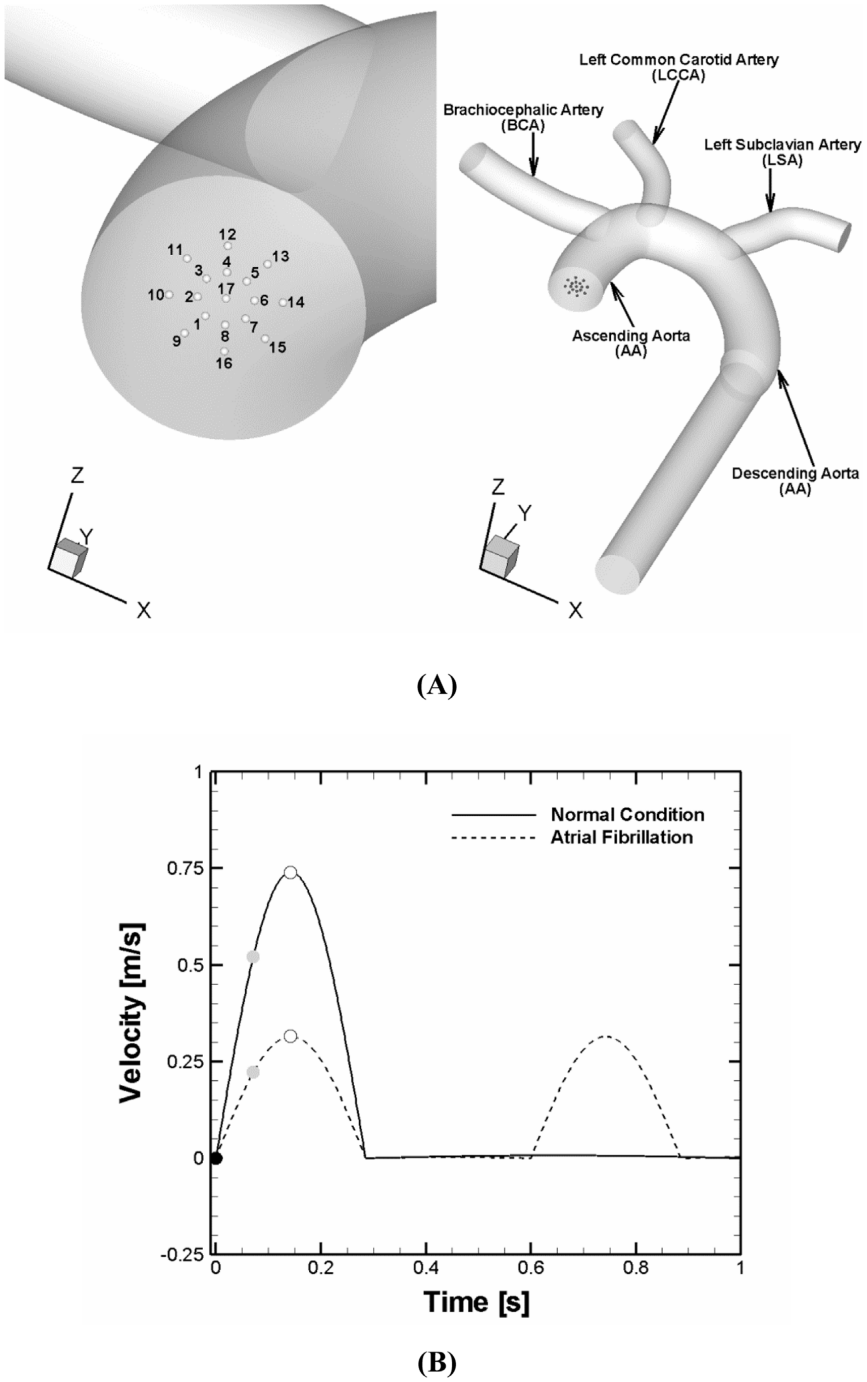
Computational domain and flow conditions for simulations. (**A**) Aortic arch model used for simulations and clot release positions at the aortic inlet. The initial positions of a clot were assumed to be circumferentially distributed at a radial distance of 0–40% of aortic radius (position 1 to 17). (**B**) Normal and AF flow velocity profiles specified at the aortic inlet. Solid and dashed lines represent normal and AF aortic flow conditions, respectively. The velocity profiles reflect the reduction in cardiac output and cycle length. The cardiac output and cycle length corresponding to the normal and AF conditions were 5.5 vs. 3.8 L/min and 1 vs. 0.6s. The black, gray, and white circle symbols denote three different clot ejection moments assumed which correspond to a beginning of systolic, accelerating, and peak stage of the cardiac cycle, respectively.

The dimension of each branch of aortic arch is variable in patients [14]. The aortic arch geometrical model used in the present study is depicted in Fig. 1A and the morphometric (diameter) parameters of the aortic arch are provided (Table 1) commensurate with the previous study of human aorta [15], [16]. All the diameters of aortic branches adopted fall within the range of aortic branch dimensions observed in the previous study [14]. In order to capture the highly dynamic flow patterns in the aortic arch, a fine mesh consisting of 1.8 million tetrahedral elements was adopted with a dense mesh distribution especially near the wall and branching regions to capture the proper boundary layer. This mesh density is consistent with the previous studies on aortic arch flow simulations [16], [17]. Also, the mesh distribution was verified to yield mesh-independent solutions; i.e., an increase to 2.7 million mesh elements did not produce a significant difference in the solution.

**Table 1:** Morphometric Parameter (Diameter) of Aortic Arch Model used for Simulations.

Location	Diameter	
	Case I	Case II
AA	29 mm	27.5 mm
BCA	19 mm	11.4 mm
LCCA	14.3 mm	7.5 mm
LSA	16.7 mm	10.1 mm

AA, BCA, LCCA, and LSA denote ascending aorta, brachiocephalic artery, left common carotid artery, and left subclavian artery, respectively.

### Computational Methods

The flow field in aortic arch and trajectory of a blood clot in the blood stream were obtained from solving the Navier-Stokes equations and Newton’s second law of motion for a particle (Appendix S1). A two-way coupling approach was adopted to appropriately describe the interaction or momentum exchange between a blood clot and flow such that not only alteration of a clot trajectory by flow but also influence of the clot properties on flow dynamics was taken into account for a wide range of clot sizes (i.e., 2–6 mm, Table 2). The finite volume commercial package ANSYS FLUENT (version 12.1, ANSYS, Inc.) was used to solve the governing equations. All simulations were performed on a Dell Workstation T7500 with two Intel Xeon processors and 12 GBytes of memory.

**Table 2:** Clot Parameters used for Simulations.

Clot Position	Clot Ejection Speed	Clot Density	Clot Diameter	Total # of Clots Tracked
1–17	0–0.8 m/s	1,080 kg/m^3^	2–6 mm	17×10×5×3 = 2550

### Flow Modeling

Hemodynamics in aortic arch often involve complex flow disturbance including turbulence. In order to account for turbulent flow behavior, k-ω SST (shear stress transport) model was adopted for the majority of simulations. For some simulations, three different flow modeling approaches (i.e., no turbulence model, LES (large eddy simulations), and k-ω SST model) were compared.

There have been various simulations of flow dynamics in an aortic arch where measured velocity profiles were imposed at the aortic inlet as boundary conditions [17]–[23]. For the present simulations, a velocity profile of a normal cardiac condition was modeled and specified at the aortic inlet such that the Reynolds number based on ascending aortic diameter reached 6500 at peak systole with a mean value of about 1200 [20]. Another velocity profile that represents an AF condition was modeled such that cardiac output and cycle length were reduced by 30% and 40%, respectively compared to normal [10]. Both velocity profiles are depicted in Fig. 1B and correspond to cardiac output of 5.5 and 3.8 L/min and Womersley number of 20 and 25.8 for the normal and AF conditions, respectively. A low inlet turbulence intensity of 1.5% was specified at the aortic inlet based on the previous studies [18], [23]. At the vessel wall, the no slip boundary condition was enforced. For the outlets, a constant fraction of the inlet flow rate with the fully-developed condition was specified at every outlet of vessels based on measured flow rates [17].

## Results

### Flow Patterns in Aortic Arch

**Figure 2:**
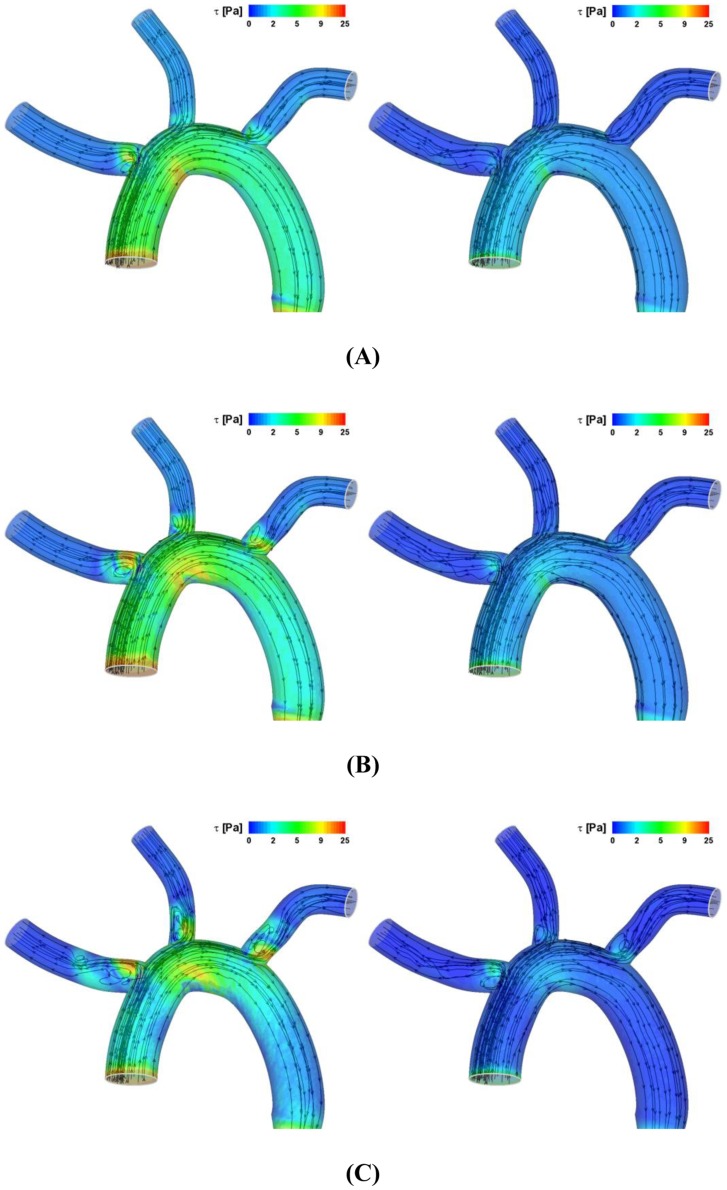
Instantaneous streamlines and WSS contour at (A) accelerating, (B) peak systole, and (C) decelerating stage of a cardiac cycle for the normal (left panels) and AF (right panels) conditions.

The hemodynamic force on the clot is a critical parameter that dictates the trajectory of clot. Since altered flow field modulates the flow force, it is important to determine how the flow field is affected by the cardiac flow condition. Figure 2 depicts the instantaneous streamlines and wall shear stress (WSS) contours at three stages of the cardiac cycle (i.e., acceleration, peak, and deceleration) for two different velocity profiles specified at the aortic inlet (i.e., normal condition and AF) as shown in Fig. 1B.

The results demonstrated that level of WSS is significantly higher for the normal than AF flow conditions considered (compare the left and right panels in Fig. 2). Disturbed flow patterns near three branching regions or branch inlets of aortic arch were shown to become intensified as the cardiac cycle changes from the accelerating to peak flow and decelerating stage of flow cycle (Fig. 2A → B → C). This was shown to be similar for both normal and AF flow conditions but more significant for the normal condition.

### Effect of Aortic Flow on Blood Clot Trajectory

To address the impact of altered hemodynamics on the clot trajectory in aortic arch, a range of blood clots (i.e., clot diameter D_clot_ = 2–6 mm) were modeled to be released at the various positions of aortic inlet shown in Fig. 1A (i.e., positions 1–17) under two different aortic flow conditions shown in Fig. 1B (i.e., normal condition vs. AF). Since the velocity of clot released is likely to be dependent on the cardiac situation, the release velocity v_release_ were assumed to be different for two different flow conditions. Also, for each flow condition, a clot was assumed to be released with two different levels of initial velocity which represent a moderate and a high speed of clot ejection from the left ventricle, respectively (i.e., V_mean_ and V_peak_ that correspond to a mean and a peak value of respective velocity profile).

**Figure 3:**
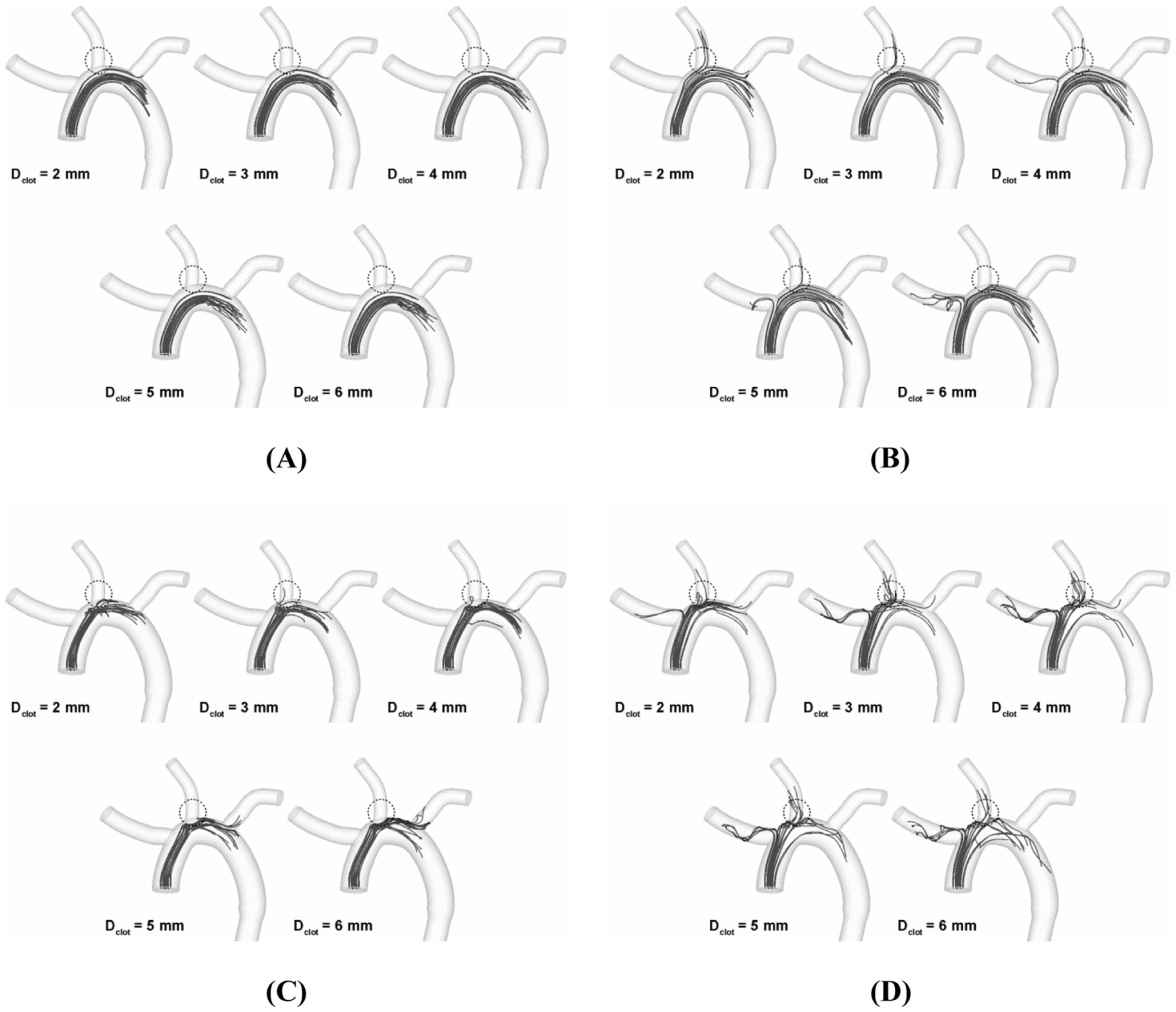
Trajectory of clots ranging from 2 to 6(A) normal and (B) AF condition, respectively.

Figure 3A depicts the trajectory of clots along the cardiac cycle of normal flow condition for the high (i.e., 0.74 m/s corresponding to the peak flow velocity) speed of clot ejection. The results showed that only a few clots considered were streamlined into various aortic branches depending on clot size. Moreover, no clots were found to be transported into any aortic branches for the moderate release speed (corresponding to the cycle-average flow velocity; data not shown). Although the alterations of clot trajectory were also observed in a similar manner for the AF flow condition, the effect was more dramatic such that the number of clots directed into the carotid was significantly increased as shown in Fig. 3B (high ejection speed corresponding to the peak flow velocity or 0.32 m/s).

### Stroke Propensity

**Figure 4:**
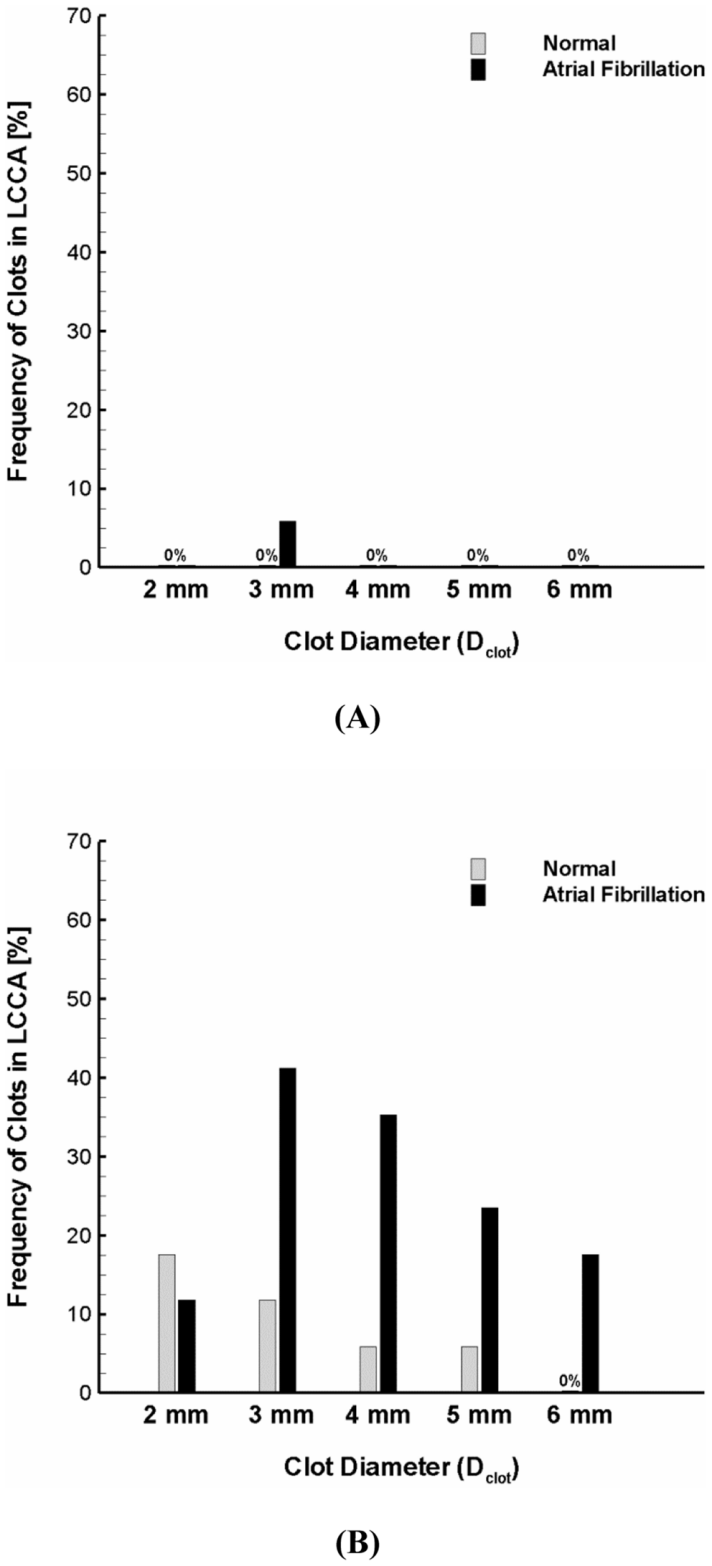
Stroke propensity for the clots of 2–6 mm diameter released at the (A) moderate and (B) high ejection speeds for the normal and AF flow conditions. The gray and black bars represent the normal and AF flow condition, respectively. Definition of stroke propensity is the percent ratio of clots whose trajectories ended up in carotid to all the clots released at the aortic inlet or 17 clots depicted in Fig. 1A.

As depicted in Fig. 3, the clot trajectory in aortic arch is regulated by hemodynamics and clot properties as well. Moreover, the altered trajectory was shown to affect the number of clots that are transported into aortic branches and in particular the carotid (dotted circle zones in Fig. 3) which may lead to embolic stroke. Figure 4 shows percentage of the clots transported to the carotid for the normal and AF.

The results suggest that only a small number of clots (i.e., 7 out of 170, ∼4%) were transported into the carotid under normal flow condition (Fig. 3A). This resulted in low propensity of stroke as indicated by the gray bars in Figs. 4A and B. Stroke propensity was shown to increase significantly, however, under AF flow conditions for various clot sizes; and especially for the high clot release speed corresponding to the peak flow velocity. Specifically, we found negligible difference in stroke propensity between the normal and AF flow condition for the moderate release velocity (compare gray and black bars in Fig. 4A) for all clot sizes considered while significant increase in stroke propensity was observed with the high release velocity (compare gray and black bars in Fig. 4B) for most clot sizes (i.e., D_clot_ = 3–6 mm).

**Figure 5:**
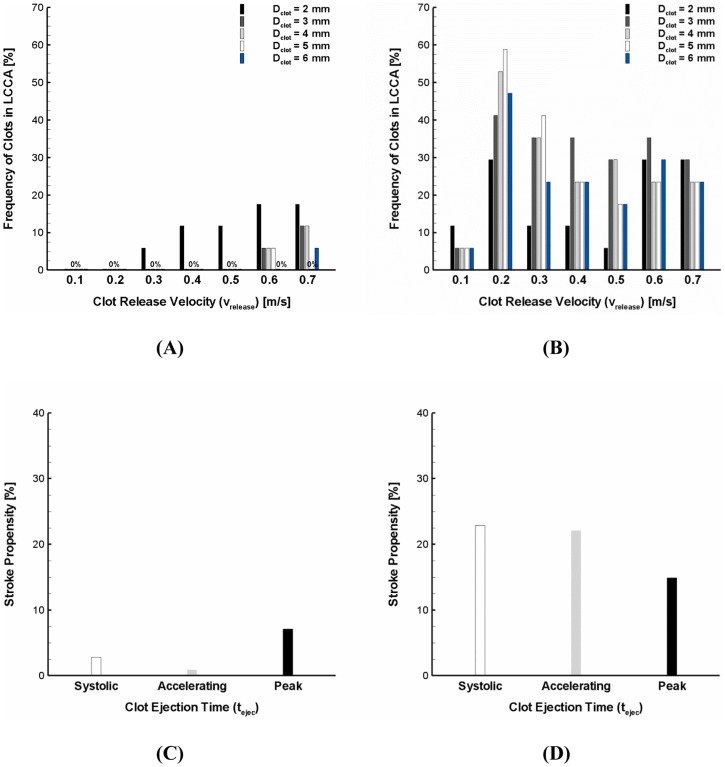
Stroke propensity for the clots of 2–6 mm diameter released at a range of clot ejection speeds (i.e., 0.1–0.7 m/s) under the (A) normal and (B) AF flow conditions. The black, dark gray, light gray, white, and blue bars represent 2, 3, 4, 5, and 6–6 mm diameter with a range of clot ejection speeds of 0–0.7 m/s released at three different ejection times indicated in Fig. 1B under the (**C**) normal and (**D**) AF flow conditions. The white, gray, and black bars denote the clot ejection moment corresponding to the beginning of systolic, accelerating, and peak stage of the cardiac cycle, respectively.

Because the speed of clot ejection from the left ventricle is entirely subject to the dynamic cardiac condition, the clot release velocity can vary widely. In order to more precisely identify the impact of flow dynamics on stroke propensity, the clots of 2–6 mm diameter were assumed to be released with a wide range of identical ejection speeds (i.e., v_release_ = 0.1–0.7 m/s) under normal and AF flow conditions. For a broad range of ejection speeds, only a small number of clots were shown to transverse into the carotid at higher ejection speeds (i.e., 0.6 and 0.7 m/s) under normal flow conditions. This resulted in virtually negligible stroke propensity for the normal flow condition over the most clot ejection speeds (i.e., v_release_ = 0.1–0.6 m/s) and clot sizes considered (i.e., D_clot_ = 3–6 mm) (Fig. 5A).

As seen in Fig. 5B, significant stroke propensity was observed over the majority of blood clot sizes and release speeds considered under the AF flow condition. Specifically, the clots were shown to result in high frequency of clot embolization in the carotid (i.e., stroke propensity of 40–60%) if they were released at a speed of 0.2 m/s which is in between the cycle-average (0.1 m/s) and peak (0.32 m/s) flow velocity under the AF condition. The larger size of clots (D_clot_ >2 mm) with a lower release speed (v_release_ = 0.1 m/s) were found to contribute much less to stroke risk.

Similar to the clot ejection speed, the time of clot ejection during the cardiac cycle is also dependent on the dynamic cardiac condition. Three different temporal points of clot ejection were assumed (i.e., a beginning of systolic, accelerating, and peak stage of the cardiac cycle, Fig. 1B) and overall stroke propensity (i.e., total frequency of clot embolization in the carotid over the clot sizes of 2–6 mm and clot ejection speeds of 0–0.7 m/s) was compared for three different ejection moments under the normal and AF flow conditions (Figs. 5C and D). The results showed that the significant increase in clot embolization frequency in the carotid is evident under the AF flow condition albeit the sensitivity of overall stroke propensity to time of clot ejection was observed for both normal and AF flow conditions.

### Effect of Aortic Arch Geometry on Stroke Propensity

**Figure 6:**
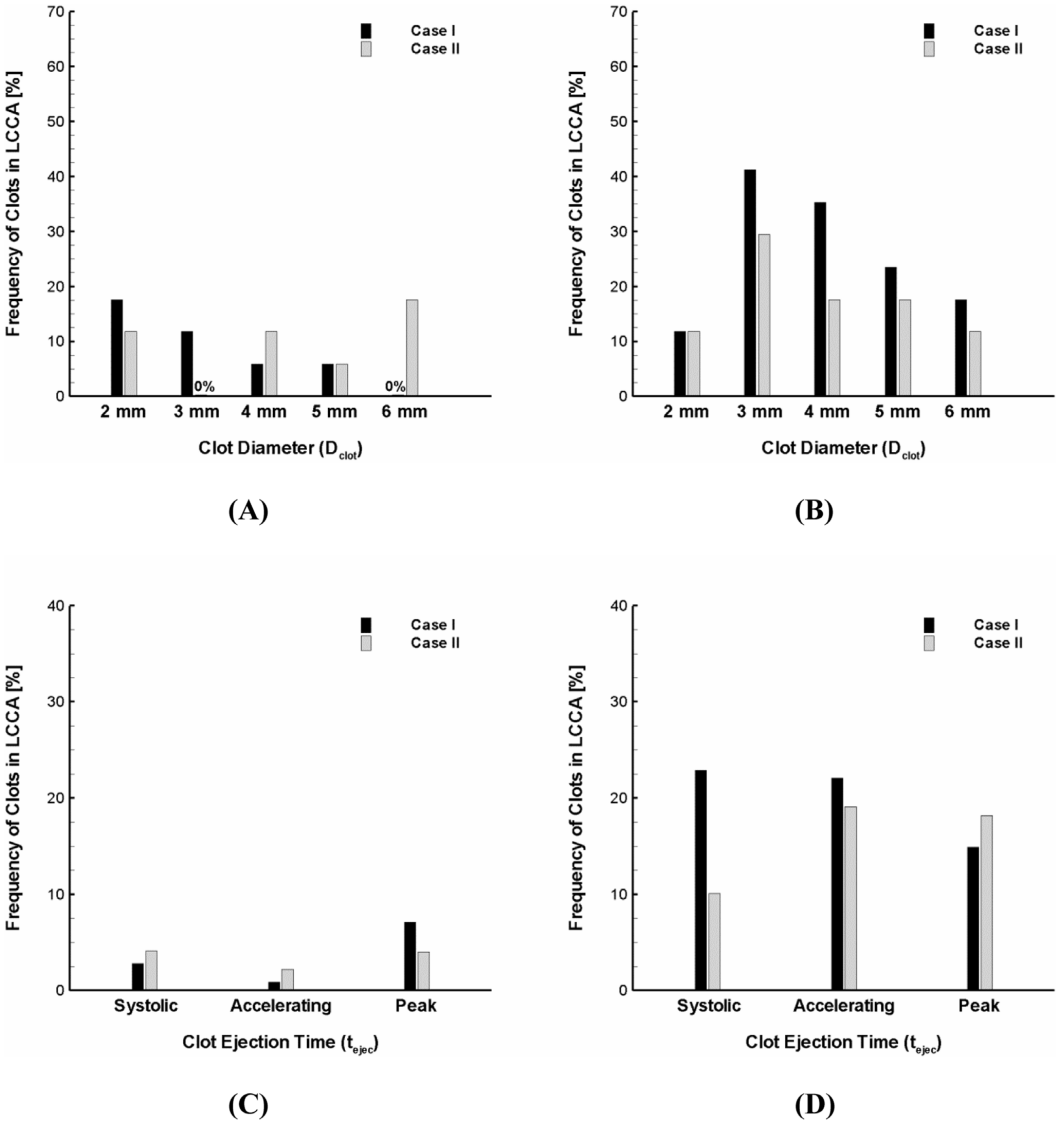
Modulation of stroke propensity for two aortic arch models under the (A) normal and (B) AF flow condition at the high ejection speed. Overall stroke propensity for the clots of 2–6 mm diameter with a range of clot ejection speeds of 0–0.7 m/s released at three different ejection points indicated in Fig. 1B under the (**C**) normal and (**D**) AF flow conditions. Case I and II indicate upper and lower ranges of carotid diameters observed in the previous study (23), respectively. Also, they represent two different branch sizes and locations relative to aorta.

The inter-individual variations in aortic arch morphology are thought to be large [14], [15]. Specifically, the diameter of carotid was shown to range from 6.9 to 14.1 mm [14]. Figure 6 shows the variation in stroke propensity under the normal and AF flow conditions for two different geometrical configurations of the aortic arch considered. The simulations showed that the frequency of clot embolization in the carotid can be significantly affected by the dimensions of aortic branches. Specifically, the stroke propensity was shown to be higher for the larger carotid diameter (Case I) than for the smaller carotid diameter (Case II) over the majority of clot sizes considered (i.e., 3–6 mm) under the AF flow condition (Fig. 6B) while the effect of carotid dimension on stroke propensity was shown to be inconsistent under the normal flow condition (Fig. 6A). Although the significant increase in frequency of clot embolization in the carotid by the AF flow condition compared to normal was not observed for the smaller carotid diameter (i.e., Case II or 7.5 mm) as evident as for the larger carotid size (i.e., Case I or 14.3 mm), the stroke propensity was shown to increase from zero up to about 30% for the clot size of 3 mm.

Sensitivity of the overall stroke propensity to the three different time points of clot ejection was also compared for two different morphological configurations of the aortic arch under the normal and AF flow conditions (Figs. 6C and D). Sensitivity of the overall stroke propensity to the time point of clot ejection was shown to be insignificant for the larger carotid dimension under the both normal and AF flow conditions. On the other hand, the results showed that variations of the overall stroke propensity depending on clot ejection moment are apparent for the smaller carotid dimension under the AF flow condition (gray bars in Fig. 6D). Despite the variability of the overall stroke propensity depending on clot ejection moment and carotid dimension, however, the general increase in stroke propensity by the AF flow condition was shown to be evident regardless of the aortic arch configurations considered (Fig. 6C → D).

### Impact of Flow Modeling on Carotid Embolization

Since flow in aorta is a complicated 3-D phenomenon, it is necessary to confirm the conclusions with various flow models. Therefore, the trajectory of clots ranging 2–6 mm of diameter with the high ejection speed was tracked for the normal and AF flow conditions by three different flow models (i.e., no turbulence model, LES, and k-ω SST model).

**Figure 7:**
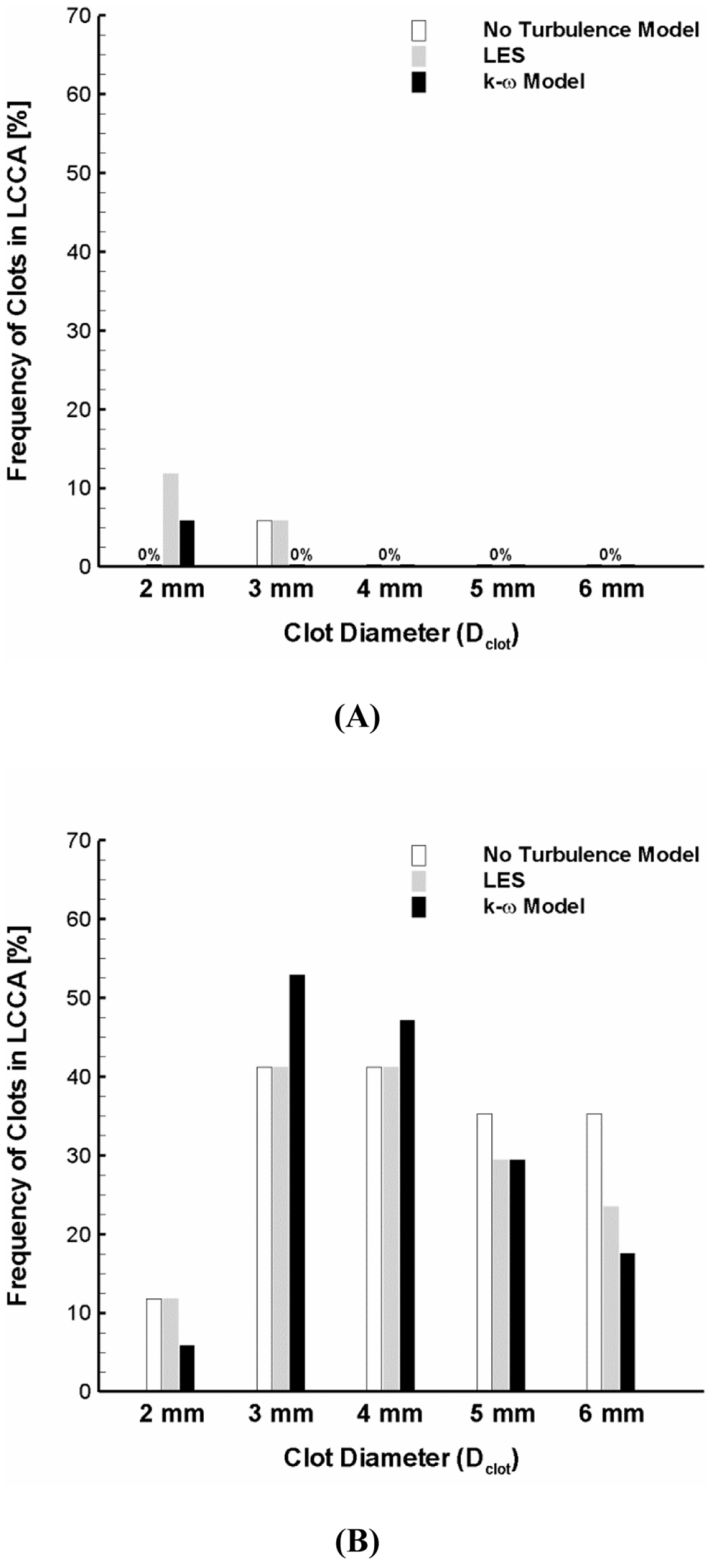
Variation of stroke propensity among three different flow modeling approaches (i.e., no turbulence model, LES, and k-ω SST model) at the high ejection speed for the normal (A) and AF (B) flow conditions. The white, gray, and black bars represent no turbulence model, LES and k-ω SST model, respectively.

Fig. 7 depicts the stroke propensity predicted by the three different flow models for the normal condition and AF. The results showed that there are some variations in stroke propensity for the 2 mm diameter clot (i.e., 0%, 11.8%, and 5.9% with no turbulence model, LES, and k-ω SST model, respectively) and for the 3 mm diameter clot (i.e., 5.9%, 5.9%, and 0% with no turbulence model, LES, and k-ω SST model, respectively) under normal flow condition. The variability in stroke propensity was shown to slightly increase for the AF condition so that the maximum difference in stroke propensity was 17.7% for the 6 mm clot diameter. Despite the variability, however, significant increase in stroke propensity induced by AF was shown to be consistent for all flow modeling considered.

## Discussion

A large number of people suffer stroke worldwide [24]. Stoke has been reported to have a high rank in cause of death in developed countries and cerebral embolism has been shown to be the cause of ischemic stroke in 40–80% of cases [25], [26]. An increase in predisposition for ischemic stroke has been identified in AF patients [3], [4]. To the best of our knowledge, there has been no study that systematically addresses how the altered hemodynamic conditions in AF can increase risk of stroke. In the present study, we performed CFD simulations to investigate flow dynamics and transport behavior of blood clots in aortic arch subject to normal and AF cardiac hemodynamic parameters. The primary finding of this study is that the flow conditions induced by AF can significantly increase the frequency of blood clots that are transported into the carotid compared to the normal aortic flow condition.

The complex 3-D geometry of the aortic arch and its branches induces complicated flow dynamics. Furthermore, AF and associated cardiac hemodynamic parameters detrimentally affect the aortic flow patterns. Since the effects of AF on cardiac hemodynamic parameters are diverse [10], the present study was primarily focused on the impact of reduction in cardiac output and cycle length in AF on the aortic flow patterns, trajectory of blood clots, and the potential for their embolic propensity in the carotid.

The flow patterns were shown to be generally similar for the normal and AF flow conditions at the aortic inlet in that the disturbed flow behavior such as flow recirculation near the branching regions of aortic arch becomes most significant as the cardiac cycle is in a decelerating stage (Fig. 2C). The level of flow disturbance and WSS was shown to be significantly higher, however, for the normal hemodynamics than the AF flow dynamics (compare the left and right panels in Fig. 2).

Discrete phase behavior of clots in continuous phase of blood flow is inevitably affected by both the release dynamics of blood clots and the aortic hemodynamics. These factors were shown to lead to a wide variety of clot trajectories as shown in Fig. 3. Specifically, the results demonstrated that the clot trajectory can be significantly altered by the clot properties such as clot size and ejection speed even under an identical hemodynamic condition. Also, the results indicate that the alterations in clot trajectory seem to be more pronounced for the AF than the normal flow condition (compare Figs. 3A and B).

In the present study, the fractions of clots that were transported into the carotid were considered as indicators of stroke propensity for the flow conditions in a normal and AF. The results demonstrated that stroke propensity is virtually negligible over the majority of clot sizes and ejection speeds considered for the normal cardiac condition (Figs. 4 and 5A). On the other hand, stroke propensity was shown to depend on clot size with broad variability at different clot ejection speeds in AF (Figs. 4 and 5B). Although stroke propensity seems to be a complex function of clot properties and hemodynamics, the results suggest that predisposition of cerebral embolism to blood clots can be significantly increased by the altered cardiac hemodynamics in AF (e.g., decrease in cardiac output and cycle length).

Aortic flow dynamics is complex and includes turbulence which is challenging to quantify. Flow dynamics in aortic arch has been investigated by various methods [16], [17], [20]–[23]. Since the trajectory of blood clot is significantly affected by hemodynamics, it is necessary to determine how blood flow dynamics and associated clot behavior can be affected by the flow model. Despite the variability of stroke propensity estimation by three different methodologies of flow modeling (i.e., no turbulence, LES, and k-ω SST model), the results confirmed that stroke propensity can be significantly increased by AF flow condition (Fig. 7).

Despite the variations in quantity of emboli depending on the properties of blood clots, flow conditions, and geometric configurations of aortic arch considered, the highest incidence of embolization occurred in the left carotid artery (Fig. 3B). This is consistent with a previous study [27] demonstrating that the majority of embolization (64%) occurred in the left cerebral hemisphere (i.e., through left carotid artery) while only 31% of emboli entered the right hemisphere (i.e., through right carotid artery).

A limitation of this study is that aortic flow pattern and the clot trajectory were investigated in idealized aortic arch models albeit they were varied to have different branch configurations (Table 1). The geometric configurations of aortic arch including aorta size, arch curvature, and branch sizes and locations are an important determinant of aortic flow field and hence the trajectory of a blood clot. Inter-individual variations in aortic arch configuration are broad and aortic flow field is not only regulated by aortic arch geometry but also by the cardiac flow condition that is strongly dependent on the cardiac dysfunction in patients.

The sensitivity of blood clot trajectory to clot property and hemodynamics (Figs. 4 and 5) and aortic structure (Fig. 6) motivates further studies on patient-specific risk assessment for stroke propensity by AF. This may enable the identification of the inter- and intra-patient stroke-prone conditions in aortic morphology (e.g., aging, anatomical variations, etc.) and cardiac dynamics (e.g., altered cardiac output, irregular heart rhythm, etc.). Specifically, since AF is characterized as dysfunction in heart rhythm including beat-to-beat irregularity in cardiac cycle duration [10]–[13], it may be interesting to investigate how clot trajectory may be affected by various heart rhythm disturbances (e.g., long diastolic pause).

This study provides a mechanistic insight on how the hemodynamic perturbations affect potential embolization of blood clots to the brain that can cause stroke. Furthermore, the current computational approach of tracking clots may be utilized to provide a basis for formulation of design principles to develop novel vascular devices that can alter blood flow field near the carotid and subclavian arteries to deflect the clots away from the brain.

## Supporting Information

Appendix S1 Numerical Methods.
(DOCX)
