# Supplementary material for: Stroke Propensity Is Increased under Atrial Fibrillation Hemodynamics: A Simulation Study
Source: PLoS One. 2013 Sep 5;8(9):e73485. doi: 10.1371/journal.pone.0073485 (PMC3764003; doi:10.1371/journal.pone.0073485)
Supplement: Appendix S1 — Numerical Methods. (DOCX) [file pone.0073485.s001.docx]

**Appendix A**

***Numerical Methods***

The flow field in aortic arch was numerically simulated by solving the continuity and momentum equations as follows:

Continuity: $\nabla\cdot\overrightarrow{V}=0$ (A1)

Momentum: $\rho\frac{\partial\overrightarrow{V}}{\partial t}+\rho(\overrightarrow{V}\cdot\nabla)\overrightarrow{V}=\nabla p+\nabla\cdot(\mu(\nabla\overrightarrow{V}+(\nabla\overrightarrow{V})^{T}))$ (A2)

Where $\overrightarrow{V}$, $p$, $\mu$, and $\rho$ are velocity, pressure, dynamic viscosity of blood, and blood density, respectively. Blood was assumed as an incompressible and Newtonian viscous fluid with a constant density of 1060 kg/m^3^ and a constant dynamic viscosity of 0.0035 kg/m-s [17]. The assumption that blood is a Newtonian fluid is consistent with the previous studies on aortic arch flow simulations [16,17,20]. The incorporation of non-Newtonian viscosity into the aortic turbulent flow model has been shown not to lead to discernible changes in flow patterns [18].

The motion of clots (discrete phase) in blood flow (continuous phase) is dictated by force balance exerted on the clot which varies dynamically with time. Since there is little data on the size distribution of AF-induced thrombi, the clot size was assumed to range from 2 to 6 mm based on the previous study [16] and clinical experience. Also, ejection dynamics of cariogenic blood clots from the left ventricle is diverse and random. Thus, a clot was assumed to be released from different locations at aortic inlet with various release velocities at different stages of a cardiac cycle (Fig. 1 and Table 2). The trajectories of the clots were tracked by solving the equation of motion for a particle (i.e., Newton’s second law of motion) as follow:

$m_{p} \frac{d\overrightarrow{V_{p} }}{dt}=\overrightarrow{F_{D} }+\overrightarrow{F_{G} }+\overrightarrow{F_{A} }$ (A3)

where $m_{p}$ and $\overrightarrow{V_{p}}$denote particle mass and velocity, respectively. The first term ($\overrightarrow{F_{D}}$) in the right-hand side of Eq. (A3) represents the hydrodynamic drag force on a particle described as follows:

$\overrightarrow{F_{D}}=\frac{1}{2}C_{D}\rho_{p}A_{p}\left|\overrightarrow{V_{S}}\right|\overrightarrow{V_{S}}$ (A4)

where $C_{D}$ is the drag coefficient, $\rho_{p}$ is the particle density, $A_{p}$ is the cross-sectional area of the particle, and $\overrightarrow{V_{S}}$ is the particle slip velocity. The second term ($\overrightarrow{F_{G}}$) indicates the gravity force. The third term ($\overrightarrow{F_{A}}$) denotes the additional forces that are virtual mass force plus additional force due to pressure gradient in the flow field. Because the drag force is dominant, similar to the previous work [16], the other possible forces such as lift force and basset force were assumed to be negligible in the present study. Interaction between thrombi and vessel wall is complex and multi-factorial including biochemical aspects as well as physical contact. Elastic collision was assumed between a blood clot and vessel wall for the current study. The clot parameters used for simulations are based on the previous works [16,28] and provided in Table 2.
